# Comparative transcriptome sequencing of tolerant rice introgression line and its parents in response to drought stress

**DOI:** 10.1186/1471-2164-15-1026

**Published:** 2014-11-26

**Authors:** Liyu Huang, Fan Zhang, Fan Zhang, Wensheng Wang, Yongli Zhou, Binying Fu, Zhikang Li

**Affiliations:** Institute of Crop Sciences/National Key Facility for Crop Gene Resources and Genetic Improvement, Chinese Academy of Agricultural Sciences, South Zhong-Guan-Cun Street 12#, Beijing, 100081 China; Graduate School of Chinese Academy of Agricultural Sciences, South Zhong-Guan-Cun Street 12#, Beijing, 100081 China

**Keywords:** Drought tolerance, Introgression line, RNA sequencing, JA and GA pathway, Co-expression network, Rice

## Abstract

**Background:**

Rice (*Oryza sativa*. L) is more sensitive to drought stress than other cereals, and large genotypic variation in drought tolerance (DT) exists within the cultivated rice gene pool and its wild relatives. Selective introgression of DT donor segments into a drought-sensitive (DS) elite recurrent parent by backcrossing is an effective way to improve drought stress tolerance in rice. To dissect the molecular mechanisms underlying DT in rice, deep transcriptome sequencing was used to investigate transcriptome differences among a DT introgression line H471, the DT donor P28, and the drought-sensitive, recurrent parent HHZ under drought stress.

**Results:**

The results revealed constitutively differential gene expression before stress and distinct global transcriptome reprogramming among the three genotypes under a time series of drought stress, consistent with their different genotypes and DT phenotypes. A set of genes with higher basal expression in both H471 and P28 compared with HHZ were functionally enriched in oxidoreductase and lyase activities, implying their positive role in intrinsic DT. Gene Ontology analysis indicated that common up-regulated genes in all three genotypes under mild drought stress were enriched in signaling transduction and transcription regulation. Meanwhile, diverse functional categories were characterized for the commonly drought-induced genes in response to severe drought stress. Further comparative transcriptome analysis between H471 and HHZ under drought stress found that introgression caused wide-range gene expression changes; most of the differentially expressed genes (DEGs) in H471 relative to HHZ under drought were beyond the identified introgressed regions, implying that introgression resulted in novel changes in expression. Co-expression analysis of these DEGs represented a complex regulatory network, including the jasmonic acid and gibberellin pathway, involved in drought stress tolerance in H471.

**Conclusions:**

Comprehensive gene expression profiles revealed that genotype-specific drought induced genes and genes with higher expression in the DT genotype under normal and drought conditions contribute jointly to DT improvement. The molecular genetic pathways of drought stress tolerance uncovered in this study, as well as the DEGs co-localized with DT-related QTLs and introgressed intervals, will serve as useful resources for further functional dissection of the molecular mechanisms of drought stress response in rice.

**Electronic supplementary material:**

The online version of this article (doi:10.1186/1471-2164-15-1026) contains supplementary material, which is available to authorized users.

## Background

Rice (*Oryza sativa* L.) is a staple food for more than half of the world’s population, especially in developing countries. Drought is the most serious environmental stress, limiting crop growth and productivity: drought-induced loss in crop yield probably exceeds losses from all other causes[1]. Drought tolerance (DT), therefore, is a major aim of rice breeding, especially in tropical Asian and African countries[2]. DT is a complex trait, and a number of quantitative trait loci (QTLs) for DT in rice have been identified[3]. However, breeding drought-tolerant rice is hard to achieve by conventional strategies, including marker-assisted selection. Understanding of the molecular mechanisms underlying DT is therefore needed for successful, knowledge-based crop improvement[4].

Determining the mechanisms directly involved in DT remains a challenging task because it involves several metabolic and morphologically adaptive pathways[5, 6]. Abscisic acid (ABA) is an important phytohormone involved in drought stress tolerance in plants, whose mechanism in plant DT is relatively clear. Under drought stress, ABA-mediated stomatal closure is the mechanism used by plants to adapt to water deficiency[7–9]. Reactive oxygen species (ROS), including hydrogen peroxide, which are widely generated under stress, have been proposed to function as second messengers in ABA signaling in guard cells[10–13]. In guard cells, ABA-stimulated ROS accumulation activates plasma membrane calcium channels and triggers stomatal closure[11, 14]. It was reported that jasmonic acid (JA) is also associated with stomatal closure under drought stress, the detailed molecular mechanisms remain elusive[15–17]. Though there is no evidence in relevance of gibberellic acid (GA) and stomatal closure under drought stress, reduction of GA levels and signaling has been shown to contribute to plant growth inhibition under several abiotic stresses, including cold, salt and osmotic stress[18, 19].

Many efforts have been made to identify the genes involved in drought stress tolerance in a number of plant species. Several drought-responsive genes encoding late embryogenesis abundant proteins, dehydration-responsive element binding protein, and protein phosphatase 2C were characterized as key components in the molecular network of DT[20]. In rice, genome-wide gene expression analyses identified many drought stress-responsive genes[21–24]. However, many of these drought stress-responsive genes have unknown functions, and details of their DT molecular mechanisms remain to be determined[20, 25].

Rice is more sensitive to drought stress than other cereals, and large genotypic variation in DT exists within the cultivated rice gene pool and its wild relatives[2]. Selective introgression of DT donor segments into a drought-sensitive (DS) elite recurrent parent by backcrossing is an effective way to improve drought stress tolerance in rice[2, 26]. In a previous study, we developed a DT introgression line (IL), H471, using the DT donor PSBRC28 (P28) and the DS recurrent parent Huang-Hua-Zhan (HHZ) (unpublished data). Compared with completely different genotypes with contrasting performance on the target trait, the ILs can largely reduce the genetic background noise in comparative transcriptomic analysis with the recurrent parent because the selected DT ILs carry a small number of genomic segments from a known DT donor[26]. Taking advantage of the combination of genome DNA re-sequencing and next-generation RNA sequencing (RNA-seq), ILs can effectively identify genes related to DT, thereby increasing our knowledge of the molecular mechanisms of this complex trait in rice[23]. In the present study, DT IL and its parental lines were used to analyze their transcriptome changes under drought stress comparatively, with the aim of extending our understanding of the genetic mechanisms of DT in rice.

## Results

### Drought stress physiology of three rice genotypes

**Figure 1:**
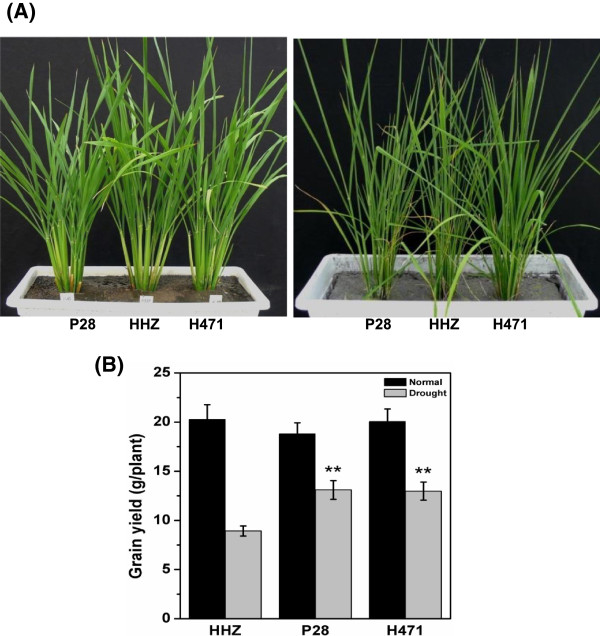
**Phenotypes of three genotypes under well-watered and drought stress conditions. (A)** Well-watered and 3 days drought stressed phenotypes of P28, HHZ, and H471 at the tillering stage. **(B)** Grain yield performance of HHZ, P28, and H471 under drought stressed and well-watered conditions. Each column represents mean ± s.d. (nine replicates); **p <0.01 versus HHZ (ANOVA, Dunnett’s multiple comparison test).

At the tillering stage, there was no visible difference observed among H471, P28, and HHZ after 1 and 2 days of drought stress; however, obvious leaf rolling in HHZ was observed after 3 days of drought stress. This phenomenon was not observed in H471 and P28 until 4 days of drought stress (Figure 1A). The yield performance of three genotypes were remarkably reduced by drought stress compared with the well-watered control; however, H471 and P28 achieved 26% and 21% higher grain yields compared with HHZ under drought conditions (Figure 1B).

**Figure 2:**
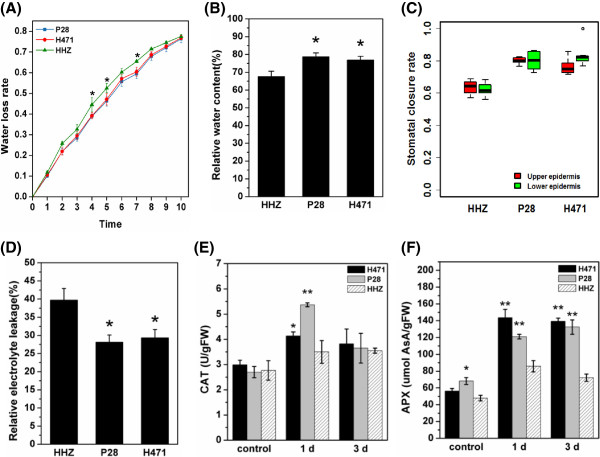
**Differential drought physiological traits of H471, P28, and HHZ. (A)** Water loss rate of P28, HHZ, and H471. For each replicate, 15 fully expanded leaves at the tillering stage were used in a triplicate experiment. **(B)** Relative water content of HHZ, P28, and H471 treated with 3 days of natural drought using the fully expanded leaves. **(C)** The stomatal closure rate in HHZ, P28, and H471 (upper epidermis: 6 randomly selected high power field for HHZ; 5 for P28; 6 for H471; lower epidermis: 8 randomly selected high power field for HHZ; 6 for P28; 6 for H471). **(D)** Relative electrolyte leakage of HHZ, P28, and H471 after 3 days of drought stress. **(E)**, **(F)** Activity of ROS-scavenging enzymes (CAT and APX) in three rice genotypes subjected to drought stress. Each column represents mean ± s.d. (three replicates); **p <0.01; *p <0.05 versus HHZ (ANOVA, Dunnett’s multiple comparison test).

To investigate the physiological difference in DT of three genotypes, several indices of drought-induced effects on leaves at the tillering stage were measured. The water loss rate (WLR) from excised leaves was determined for the three genotypes: H471 and P28 showed relatively lower WLR than HHZ over a period of 10 h (Figure 2A). Accordingly, the relative water contents (RWCs) in H471 (76.9%) and P28 (78.8%) were significantly higher than that in HHZ (67.6%) after 3 days of drought stress (Figure 2B).

Stomatal closure is one of the first responses to drought conditions that might control plant dehydration[27]. To investigate the stomatal status of the three rice genotypes at the tillering stage under drought stress, leaf surfaces were examined using scanning electron microscopy (SEM). H471 and P28 had significantly higher stomatal closure rate than HHZ after 1 day of drought stress (*p* <0.01, Kruskal-Wallis ANOVA, Dunn’s multiple comparison test) (Figure 2C). Relative electrolyte leakage (REL), which is an indicator of cell membrane injury, was also detected. The RELs of H471 and P28 were significantly lower than that of HHZ after 3 days of drought stress (Figure 2D), indicating that H471 and P28 experienced significantly less cell membrane injury than HHZ under drought stress conditions. Additionally, the activities of catalase (CAT) and ascorbate peroxidase (APX) in H471 and P28 were significantly higher than that in HHZ under drought stress conditions (Figure 2E and F), showing their active detoxification by reactive oxygen scavenging regulation in the two genotypes in response to drought[28]. Taken together, these results demonstrated that the DT of IL H471 was significantly improved over that of HHZ, reflecting the introgression of favorable alleles from DT donor P28 into the HHZ background.

### Global gene expression profiling of three genotypes under well-watered and drought stress by transcriptome sequencing

At the tillering stage, total RNA from leaves of HHZ, P28, and H471 at 1 and 3 days of drought stress and its corresponding well-watered control were paired-end sequenced using Illumina sequencing technology. A total of 17.8–23.5 million reads of 100 bp in length were generated for each sample, and the number of mapped reads were in the range of 14.4–19.6 million (Table 1). The unique matching ratio was in the range of 77.7–79.7% (Table 1), the unique matching reads were used for further analysis. The high-quality reads from individual libraries were mapped to the rice genome; more than 22,774 mapped genes per library were determined simultaneously.

**Table 1:** **Mapping results of RNA-Seq reads of HHZ, P28 and H471 under 1d and 3d drought stress and control conditions**

Samples	Total filtered pair-end reads	Total mapped reads (%)	Unique mapped reads (%)	Total mapped genes
HHZ-ck 1	2 × 8,894,670	14,403,823 (81.4)	13,766,465 (77.8)	23129
HHZ-ck 2	2 × 9,326,860	15,241,546 (81.8)	14,547,450 (78.0)	
P28-ck 1	2 × 10,384,097	16,978,445 (81.9)	16,233,203 (78.3)	23693
P28-ck 2	2 × 10,326,754	16,811,305 (81.4)	16,047,198 (77.7)	
H471-ck 1	2 × 10,068,639	16,551,528 (82.2)	15,823,734 (78.6)	23625
H471-ck 2	2 × 10,083,264	16,585,631 (82.3)	15,852,617 (78.6)	
HHZ-1d 1	2 × 11,727,199	19,581,596 (83.5)	18,660,210 (79.6)	23078
HHZ-1d 2	2 × 9,958,934	16,628,254 (83.5)	15,864,006 (79.7)	
P28-1d 1	2 × 11,594,160	19,305,500 (83.3)	18,463,197 (79.7)	22774
P28-1d 2	2 × 10,484,144	17,435,333 (83.2)	16,669,090 (79.6)	
H471-1d 1	2 × 9,853,652	16,126,140 (81.9)	15,390,608 (78.1)	23320
H471-1d 2	2 × 10,868,092	17,847,671 (82.2)	17,055,619 (78.5)	
HHZ-3d 1	2 × 10,071,423	16,584,356 (82.4)	15,857,639 (78.8)	22976
HHZ-3d 2	2 × 10,042,756	16,586,139 (82.6)	15,844,907 (78.9)	
P28-3d 1	2 × 10,271,850	16,943,591 (82.5)	16,175,470 (78.8)	23017
P28-3d 2	2 × 10,093,202	16,568,958 (82.1)	15,817,307 (78.4)	
H471-3d 1	2 × 10,087,890	16,669,962 (82.7)	15,912,430 (78.9)	22974
H471-3d 2	2 × 9,948,863	16,440,601 (82.7)	15,735,994 (79.1)	

Note: ck indicates well watered control; 1d and 3d indicate the drought treatment time; 1 and 2 in the first row indicate two replicates of each sample.

The detected expressed genes in all samples were subjected to cluster analysis. As shown in Additional file1, the three genotypes under 1 and 3 days of drought stress and control conditions were separated from each other: H471 and HHZ were clustered together in each condition subgroup, which was consistent with their similar genetic background. Based on this result, the transcriptomic response to 1d and 3d drought stress could be classified as mild stress response (MR) and severe stress response (SR), respectively, in consistent with the previous reports on abiotic stress gene profiling[29, 30].

Correlation analysis was used to assess the quality of Illumina sequencing results between two replicates of each sample. The results indicated that the coefficient of correlation between the biological samples was high, supporting the reproducibility of the results (Additional file2). To validate the Illumina sequencing results, quantitative real-time reverse transcription-PCR (qRT-PCR) was used to assess the expression levels for 27 genes of rice independently. The genes and primer sets used are shown in Additional file3. RNA samples extracted from three additional replicate sets were used as templates. The high correlation (*R*^*2*^ = 0.93, *p* <0.01) between RNA-seq and qRT-RCR expression values indicated that there was a good agreement between both approaches (Additional files4A and4B).

### Intrinsic transcriptome differences of H471 and P28 compared with HHZ prior to drought stress

Phenotypic differences resulting from gene expression variation have been observed in species[30, 31]. To investigate the intrinsic differences in gene expression between the DT genotypes and the DS genotype, the gene expression levels in H471 and P28 were compared with that in HHZ under control conditions. Between P28 and HHZ, 1282 genes were identified as differentially expressed, which is consistent with their large genetic difference. However, there were only 343 genes detected to be differentially expressed between H471 and HHZ under normal growth conditions (Additional file5). This is consistent with only a few of chromosome fragments being introgressed from P28 to HHZ. The genes with higher basal expression level in H471 compared with HHZ were mainly functionally enriched in oxidoreductase activity, lyase activity, carboxylic acid metabolic process, response to stress, and cofactor binding (Additional file6–1).

These constitutively differentially expressed genes in P28 and H471 compared with HHZ under control conditions could be classified into two groups based on their expression patterns under drought stress conditions. The first group comprised those that were unresponsive to drought stress, including 503 and 74 genes from P28 and H471, respectively, which are functionally enriched in redox regulation and apoptosis-associated proteins (Additional file7–1 and7–2). The second group comprised 779 and 269 genes differentially expressed in P28 and H471 after at least 1 or 3 days of drought stress (Additional file7–3 and7–4). Among them, 67 genes (Additional file7–5) with higher basal expression in both P28 and H471 were functionally enriched in oxidoreductase and lyase activity. Further analysis indicated that 10 of these genes were colocalized in the introgressed regions, implying their positive role in the response to drought stress.

### Comparative transcriptome profiling of three genotypes under drought stress

To determine the similarities and differences in drought-induced transcriptomes in the three genotypes, the gene expression alterations in the three genotypes under 1 and 3 days of drought stress compared with their respective controls were analyzed. In H471, P28, and HHZ, 7862, 7717, and 7625 DEGs were identified, respectively, after 1 or 3 days of drought stress.

**Figure 3:**
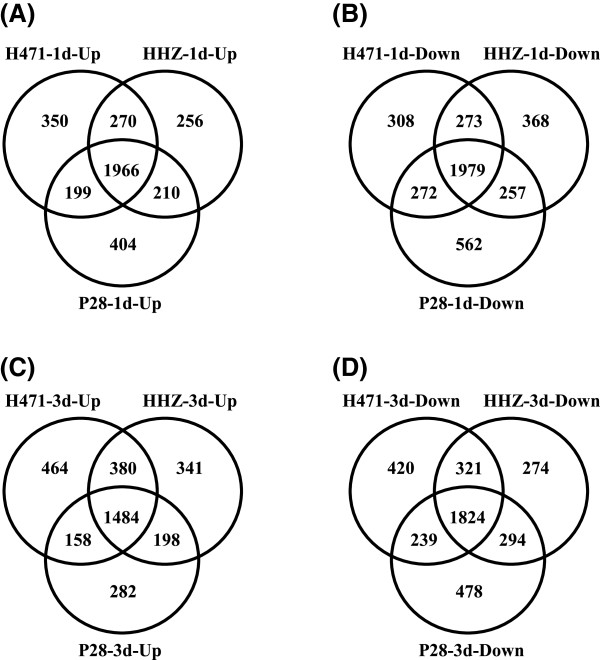
**Venn diagram of differentially expressed genes (DEGs) in H471, HHZ, and P28 under 1 and 3 days of drought stress.** DEGs were identified with adjusted *p*-value <0.001. **(A)**, **(B)**, **(C)**, and **(D)** show the Venn diagram results for the three genotypes of 1 day up-regulated, 1 day down-regulated, 3 days up-regulated, and 3 days down-regulated genes, respectively, under drought stress.

After 1 day of drought stress, there were 5617, 5849, and 5579 DEGs detected in H471, P28, and HHZ, respectively. Venn diagram analysis indicated that 3945 DEGs (1966 up-regulated and 1979 down-regulated) were commonly regulated in the three genotypes by 1 day of drought stress (Table 2, Figure 3A and B) which accounted for 70.23%, 67.45% and 70.71% of total DEGs in H471, P28 and HHZ, respectively, indicating that the mild drought stress response of the different genotypes is mostly the same. GO analysis showed that the commonly up-regulated genes were functionally enriched in the regulation of the biological process, signaling process, and carbohydrate metabolic process (Additional file6–2); while the shared down-regulated genes in the three genotypes were mainly associated with cellular protein metabolic process, phosphate metabolic process, and transport (Additional file6–3).

**Table 2:** **Summary of differentially expressed genes in H471, HHZ and P28 under 1d and 3d drought stress compared with its respective well-watered control**

	1d			3d		
	Up-regulated	Down-regulated	Sub-total	Up-regulated	Down-regulated	Sub-total
H471	2785	2832	5617	2486	2804	5290
P28	2779	3070	5849	2122	2835	4957
HHZ	2702	2877	5579	2403	2713	5116
common	1966	1979	3945	1484	1824	3308

After 3 days of drought stress, 5290, 4957, and 5116 genes were identified as DEGs in H471, P28, and HHZ, respectively (Table 2), showing relatively fewer DEGs could be detected at the SR phase compared with the MR phase. Among them, 3308 DEGs (1484 up-regulated and 1824 down-regulated) were commonly regulated in three genotypes at the SR phase which accounted for 62.53%, 66.73% and 64.66% of total DEGs in H471, P28 and HHZ, respectively (Figure 3C and D). GO analysis of these DEGs indicated that carbohydrate metabolic process, regulation of cellular process, transcription, macromolecule modification, cellular protein metabolic process, and transport were highly enriched in the SR DEGs (Additional files6–4 and6–5). Taken together, the results showed that broad functional categories of genes were commonly involved in drought stress response.

### Effect of introgression on the transcriptome of H471 in response to drought stress

The backcross introgression strategy is widely used for crop improvement. The introgressions combine the genetic background of the recurrent parent, which could result in novel changes in gene expression. To evaluate the effect of introgression on transcriptome of H471 under drought stress, the genome-wide gene expressions in H471 and HHZ under drought stress were compared. The results indicated that 460 and 380 genes were up- and down-regulated, respectively, in H471 compared with HHZ under drought stress (Additional file8). Only a small proportion of the DEGs (128 up-regulated, 103 down-regulated) colocalized with the introgressed regions (Additional file8), indicating introgression could contribute to new DT expression phenotypes in H471 relative to HHZ.

GO enrichment analysis of these DEGs highlighted the following functional categories: signaling transduction, transcription regulation, stress response, hormones (GA, JA) signal transduction, and ROS homeostasis (Additional file9).

*Genes related to signaling transduction:* 18 genes encoding receptor kinases (RKs) were differentially expressed in H471 *vs*. HHZ under drought conditions. Among them, two genes encoding cysteine-rich receptor-like protein kinases (RLKs) (LOC_Os07g43560, LOC_Os07g43570) were up-regulated in H471 *vs*. HHZ and one was repressed (LOC_Os04g30040). Eight genes encoding leucine-rich repeat transmembrane protein kinases were up-regulated and five were down-regulated in H471 relative to HHZ. Additionally, 26 genes encoding kinase proteins were differentially expressed in H471 *vs*. HHZ under drought stress. These included four genes encoding calmodulin-dependent protein kinases (CDPKs) (LOC_Os03g20380, LOC_Os03g43440, LOC_Os05g26870, and LOC_Os10g39420). Three genes encoding EF hand calcium-binding proteins (OsCML15, OsCML18, and OsCML31) were up-regulated in H471, and two genes encoding CaLB domain proteins (LOC_Os08g20544, LOC_Os02g27130) were down-regulated in H471 compared with HHZ under drought stress, indicating their different roles in the drought stress response.

**Figure 4:**
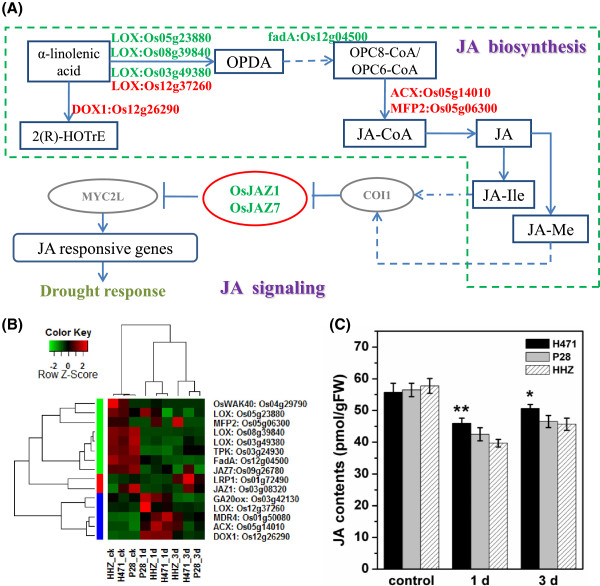
**Jasmonic acid (JA) related pathways may play key roles in drought tolerance in H471. (A)** JA synthesis and signaling pathways that contain differentially expression patterns between H471 and HHZ under drought conditions. Gene names in red and green color indicate up- and down-regulated in response to drought stress, respectively. **(B)** Hierarchical clustering of JA-related genes and some co-regulated genes. The color scale indicates the expression value. **(C)** JA contents in H471, HHZ, and P28 under drought conditions. Each column represents mean ± s.d. (three replicates); **p <0.01; *p <0.05 versus HHZ (ANOVA, Dunnett’s multiple comparison test).

*Phytohormone related proteins*: 16 genes encoding ten JA and six GA related proteins were differentially expressed in H471 *vs*. HHZ under drought stress (Additional file9). Kyoto encyclopedia of genes and genomes (KEGG) analysis of these DEGs showed that alpha-linolenic acid metabolism (ko00592), which is associated with JA synthesis, was highlighted in H471 (Additional file10). Eight JA biosynthesis-related genes, encoding four lipoxygenase (LOX), one alpha-dioxygenase (DOX1), one acyl-CoA oxidase (ACX), one enoyl-CoA hydratase/3-hydroxyacyl-CoA dehydrogenase (MFP2) and one acetyl-CoA acyltransferase (fadA), were differentially expressed in H471 compared with HHZ under drought stress. Meanwhile, two genes (*OsJAZ1* and *OsJAZ7*) involved in the JA signaling pathway were also significantly up-regulated in H471 *vs*. HHZ under drought stress. Consistently, the JA content in H471 was significantly higher than that in HHZ under drought stress, even though the JA contents in all three genotypes were repressed by drought compared with their respective control (Figure 4, Additional file11). A GA biosynthesis-related gene, encoding putative gibberellin 20-oxidase 2 (GA20ox2) was significantly up-regulated after 1 day of drought stress, while a GA deactivation-related gene encoding gibberellin 2-oxidase was evidently up-regulated after 3 days of drought stress in all genotypes. Importantly, four putative gibberellin receptor encoded genes were detected to be differentially expressed in H471 compared with HHZ under drought stress. These results indicated that the JA and GA pathway is involved in drought stress tolerance in the DT IL H471.

*Transcription regulation related genes*: 36 transcription factor (TF) genes were differentially expressed between H471 and HHZ under drought stress. Among them, three AP2/EREBP genes (LOC_Os01g64790, LOC_Os03g08470, LOC_Os04g57340) were significantly up-regulated in H471, which were previously identified to be highly involved in drought and salt stress[32]; *OsWRKY4* (LOC_Os01g53040) and *OsWRKY9* (LOC_Os01g18584) were highly expressed in H471 relative to HHZ under drought; these two genes were associated with biotic stress tolerance in a previous study[33, 34]. The others included four MYB TFs, five bHLH TFs, and seven NAC proteins, most of which were up-regulated in H471 compared with HHZ under drought, implying their positive role in the drought stress response.

*Genes encoding redox regulation-related proteins*: A set of 19 genes related to redox regulation were enriched in H471 compared with HHZ under drought stress. These genes included those encoding ascorbate peroxidase, oxidoreductase, peroxidase precursor, glutathione S-transferase, and glutathione synthetase (Additional file9), showing that redox regulation is involved in the molecular mechanisms of drought stress tolerance.

*Genes related to carbohydrate metabolism and osmotic adjustment*: 9 genes encoding UDP-glucoronosyl and UDP-glucosyl transferase (UGT) family proteins and genes encoding three osmotins and one malate synthase were differentially expressed in H471 *vs*. HHZ under drought. These genes were previously reported to be involved in sugar metabolism and detoxification[35, 36] and osmotic adjustment[37].

### Co-regulatory gene networks of H471 in response to drought stress

**Figure 5:**
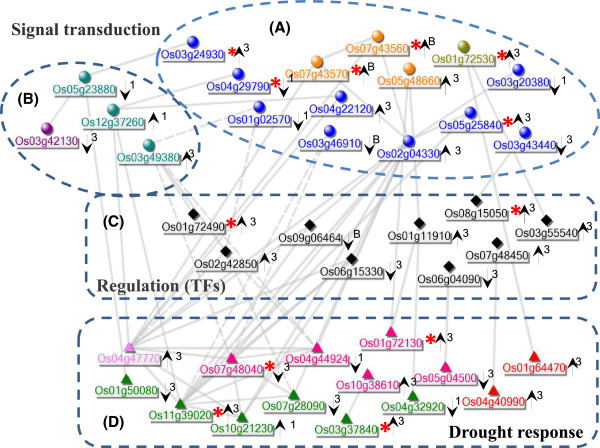
**Co-regulatory networks of genes differentially modulated in H471 under drought stress (false discovery rate 0.05).** Co-regulation analysis was based on the calculation of pairwise Pearson correlation coefficient (PCC) of logarithmic expression values (with a cutoff of 0.75) in Rice Oligonucleotide Array Database. Four subgroups, indicated with letters from **A**–**D** which also distinguished by shapes, were identified as their putative functions. Different colors indicate genes with unique function class: olive, Ca2+ signal related; gold, receptor kinase; blue, kinase; violet, TF; green, K + transporter; Pink, redox regulation; black, osmotin; red, carbohydrate metabolism. The thickness of the edges is proportional to the PCC. Asterisks indicate the genes located in the introgression segment from P28. Arrows indicate up- and down-regulated in response to drought stress, respectively; and 1, 3 and B with arrows show 1 day, 3 days, both 1 and 3 days drought stress, respectively.

To explore the genetic networks associated with DT, all DEGs belonged to the above function categories in H471 compared with HHZ under drought stress (Additional file9) were subjected to co-expression network analysis. Forty of them were found to be co-regulated and formed a complex network (Figure 5). In this network, the genes could be separated into four groups according to their putative functions. Group A was enriched in stress signaling transduction, including 13 genes encoding receptor kinases, protein kinases, and Ca^2+^ related protein. Four genes in group B were functionally involved in hormone signaling pathways: three genes encoding lipoxygenases (LOXs) and one gene encoding GA20ox2; LOX is involved in JA biosynthesis and signaling[38, 39]; and GA20ox2 is related to the biosynthesis of gibberellin[40]. The genes in group C were enriched in transcription regulation, including two NAC, one MYB, three CCT/B-box zinc finger proteins, and one HLH type TFs. The group D genes were mainly involved in ion transport, including two high affinity K^+^ transporter 5 s, three ABC transporters, and an ATP synthase subunit C family protein; and redox homeostasis, including peroxidase superfamily proteins, two glutathione S-transferases, and one short-chain dehydrogenase/reductase; and carbohydrate metabolism, including UDP-glycosyltransferase and malate synthase. These co-regulated genes in group D were evidently downstream in the drought stress tolerance mechanism of H471.

### Colocalization of DT related DEGs in the introgression fragments and QTL intervals

**Figure 6:**
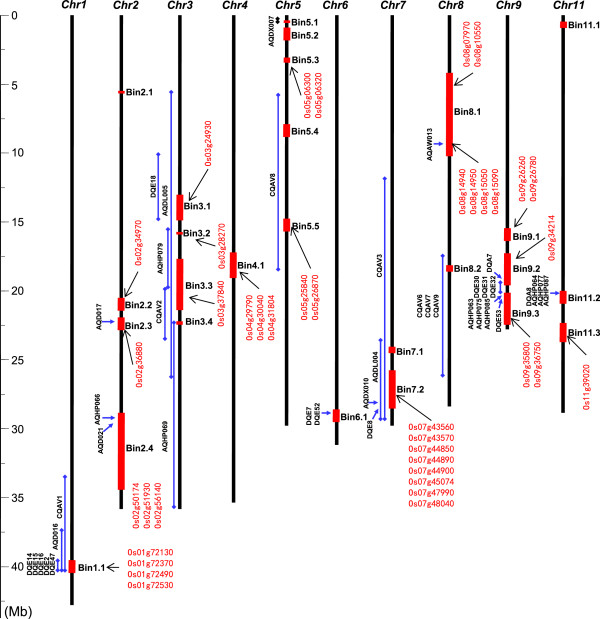
**The recombination map of H471 and drought stress-related quantitative trait loci (QTLs) located in or near the introgressed regions.** Names of QTLs, differentially expressed genes and introgressed bins are shown on the sides of the chromosome, more details are provided in Additional file12.

According to the results of genotyping by resequencing, 26 P28 fragments were introgressed into H471 (Figure 6). We analyzed the previously reported DT-related QTLs based on the Gramene QTL database (http://archive.gramene.org/qtl/) compared with the introgression intervals in H471. The results indicated that 39 known DT QTLs were co-localized in the introgressed regions in H471 (Figure 6).

Correspondingly, all DEGs, including those differentially expressed in H471 compared with HHZ under control, 1 day or 3 days of drought stress conditions, were mapped onto the rice chromosome: 289 of these DEGs were localized on the introgressed intervals. Furthermore, 205 DEGs were localized onto 15 DT-related QTL intervals that overlap with the introgressed fragments in H471 (Additional file12). Seven genes on the introgressed region of chromosome 1 were detected to be significantly up-regulated in H471 relative to HHZ after 3 days of drought stress: bHLH TF, lateral root primordium (LRP) protein, beta-1,3-glucanase 3, glycosyl hydrolase, glutathione S-transferase, and bidirectional amino acid transporter. Several drought-related QTLs, including those for leaf rolling, osmotic adjustment, and relative water content[41–43] were colocalized on this region; thus, up-regulation of these seven genes in H471 could be positively related to physiological and metabolic adaptation to drought stress. There was also a cluster of 10 genes located on the introgressed segment of chromosome 4 that had no corresponding DT QTLs. These genes included eight wall-associated kinase (WAK) family proteins and two cysteine-rich RLKs were found to be constitutively down-regulated in H471 compared with HHZ. It was evidenced that WAKs and cysteine-rich RLKs are essential for the normal regulation of cell enlargement and abiotic stress sensing[44, 45], reduced expression of these genes in H471 might negatively affect cell growth and the stress response.

## Discussion

### The DT IL showed enhanced drought stress tolerance

ILs have been widely used in genetic analysis and molecular breeding[26, 46]. Drought-selected ILs have provided a useful genetic resource to improve our understanding of the genetic and molecular basis of DT in rice[2]. In the present study, the DT IL H471 and donor P28 were more tolerant to drought stress than the recurrent parent HHZ. Under drought stress, H471 and P28 showed delayed leaf rolling, lower RWL, higher RWC, higher stomatal closure rate, relatively lower cell membrane injury, and with significantly higher CAT and APX activities than HHZ, indicating that H471 and P28 underwent complex morphological and physiological changes to resist drought stress[47]. These results showed that H471 and P28 used a typical DT strategy to cope with drought stress by limiting water loss and the enhanced DT of H471 over HHZ was caused by introgression of favorable alleles from donor P28.

### Genome-wide transcriptome reprogramming of H471 and its parental lines in response to time-series drought stress

A comparative transcriptome analysis of three genotypes under 1- and 3-day drought stress indicated that the DT and DS genotypes shared a large proportion of drought-induced DEGs, revealing common drought stress-responsive processes. GO analyses showed that commonly up-regulated genes at the MR phase were highly enriched in signaling transduction and transcription regulation, consistent with previous reports that abiotic stress triggered the MR phase of transcriptome alterations of genes related to signaling cascades, including receptor kinases, transcription factors and components of calcium signaling[30, 48]. However, after 3 days of drought stress, the common DEGs were mainly involved in carbohydrate metabolic process, post-translational protein modifications, transport, and redox regulation, implying that downstream genes related to metabolite adaptation, ROS homeostasis, and post-transcriptional regulation were involved in the severe drought stress response, in accordance with previous results[48, 49].

### Effect of introgression on the transcriptome in response to drought stress

The DEGs on the introgressed regions in H471 relative to its recurrent parent HHZ could have direct effects on its DT phenotype changes. Identification of those DEGs co-localized with identified DT-related QTLs provides useful data for DT molecular breeding and gene functional dissection. Meanwhile, it was reported that donor introgressions combine recurrent parental alleles that might result in novel changes in expression, and many genes showing non-parental expression patterns were identified outside of the introgressed fragments[50]. In this study, a set of genes in the DT introgression line H471 were exclusively differentially expressed compared with its parental lines, especially under drought stress, showing a unique expression related to the DT phenotype. These expression changes beyond the introgression regions might correspond to the well-known transgressive or nonparental expression in hybrid crop plants[51]. However, it was determined that the genome-wide expression changes in an IL might result from activation of transposon *mPing*[52] or transgressive siRNA[53]. The molecular mechanisms of this non-parental expression alteration, especially under drought stress, need to be further elucidated.

### A complex genetic network including the JA and GA pathway is involved in drought stress tolerance

Drought stress tolerance is a complex trait and involves many genes. Hence, deciphering the molecular mechanisms underlying DT in plants is a challenging task. Genome-wide identification of drought-responsive regulons in contrasting DT genotypes with similar genetic backgrounds could help to dissect novel genetic components involved in DT. We comparatively analyzed the differential gene expression between H471 and HHZ under drought stress: 840 genes were differentially expressed, indicating substantial transcriptome reprogramming in H471 under drought stress compared with HHZ.

A set of genes related to signaling transduction including RKs, CDPKs, and CBPs were up-regulated in H471 compared with HHZ. RLKs play an important role in plant growth and responses to abiotic stresses by activating initial signaling transduction[45]; CDPKs and CBPs are the main components in the stress signaling pathway, which acts by modulating ABA signaling and reducing the accumulation of ROS[54, 55]. Up-regulation of these genes in H471 implied their positive role in DT by enhancing signaling pathways under drought stress.

Plant hormones, including ABA, GA and JA, play key roles in their ability to adapt to changing environments[15, 18, 19, 56]. GO and KEGG analyses revealed that genes related to JA biosynthesis and the signaling pathway were differentially expressed in H471 relative to HHZ under drought stress, implying that JA was involved in drought response. JA plays an important role in plant growth, development and stress response[57]. The JA signaling and biosynthesis genes were found to be significantly regulated by drought in *Arabidopsis*[58] and rice[59]. In this study, the JA levels in all three genotypes were repressed by drought compared with their control, this result indicated that JA is probably not required at high concentration under drought stress, and an increase in JA content might negatively affect plant growth as suggested by previous reports[58, 60]. However, the JA content was significantly higher in H471 than in HHZ and P28 under drought stress, which correlated with the increased expression of JA biosynthesis genes in H471 relative to HHZ, demonstrating that JA plays an important role in DT. Previous studies indicated that JA affected the transcript levels of genes related to antioxidants under water stress[61]; relative higher JA content in H471 might enhance drought tolerance by modulating antioxidant homeostasis.

It was shown that JA could interact synergistically and antagonistically with other phytohormones[60, 62]. In this study, differentially expressed GA-related genes in H471 compared with HHZ under drought stress provide evidence for the role of GA metabolism and the regulation of the GA signaling pathway on exposure to drought stress which consistent with latest study. Emerging evidence for interaction of the GA-signaling molecule DELLA with components of the signaling pathway for the stress hormone JA[19] suggests that GA signaling might integrate multiple hormone signaling pathways in response to drought stress, however the crossroads of these interactions still remain to be elucidated.

TFs are hub regulators in response to biotic and abiotic stresses. Several TFs, including AP2/EREBPs, WRKYs, bHLHs, and NACs, were highly up-regulated in H471 compared with HHZ under drought stress. All these TFs were previously reported to be involved in transcription regulation of abiotic stresses tolerance[20, 63]. Importantly, AP2/EREBPs, WRKYs, and NACs are key regulators of ABA-mediated stomatal closure and, hence, drought responses[64, 65]. bHLHs play an important role in the JA-mediated regulatory network of the abiotic stress response[66]. Differential expression of these TFs in H471 relative to HHZ implied that both ABA and JA play a central role in regulating drought stress tolerance in H471.

Co-expression analysis showed that there were core DT genes forming a complex network in H471 (Figure 5), including genes related to signaling transduction, the JA pathway, and TFs, and those involved in downstream functions, such as carbohydrate metabolism, ion transport, and ROS regulation. In the network, a bHLH TF (LOC_Os01g11910) is connected to a receptor-like kinase (LOC_Os05g48660) and a hub 5-AMP-activated protein kinase (LOC_Os02g04330), interacts with glutathione S-transferase (LOC_Os01g72130), LZ-NBS-LRR protein (LOC_Os11g39020), and peroxidase (LOC_Os07g48040), and is then further connected with two JA biosynthesis lipoxygenases (LOC_Os03g49380 and LOC_Os12g37260). All these genes form a complex interacting network. However, their real interactions need to be confirmed by further experiments, including yeast-two-hybridization screening. These co-expressed gene networks provide useful information for dissecting the molecular mechanisms underlying drought stress tolerance in rice.

## Conclusions

In this study, a DT IL and its parental lines, including the DT donor and DS recurrent parent, were used to characterize the differences of leaf transcriptome dynamics under 1 and 3 days of drought stress at the tillering stage using high-throughput RNA sequencing. Drought induced transcriptome reprogramming in a DT IL H471 could result from introgressed chromosome segments from the DT donor P28, and the differentially expressed genes in the H471 relative to the HHZ under drought stress might contribute to the enhanced drought tolerance, finally improved yield performance under stress. Co-expression analysis revealed a complex regulatory network, including genes related to the signaling transduction, JA and GA pathways, transcription regulation, redox control and osmotic adjustment, involved in drought stress tolerance. The data obtained in this study could extend our understanding of the molecular mechanisms of DT in rice.

## Methods

### Plant materials and experimental treatments

Three rice genotypes were used in this study. HHZ is a widely used *indica* inbred rice in South China, with high yield and good quality, which is sensitive to drought stress. P28 is an *indica* rice variety from the Philippines and is well adapted to drought stress. H471 is a BC_1_F_5_ drought-tolerant IL with a few chromosomal fragments introgressed from the donor parent P28 into background of the recurrent parent HHZ. Genome-wide single nucleotide polymorphism analysis by re-sequencing showed that H471 differs from HHZ at 26 genomic segments from P28, with sizes in the range of 57–6057 kb (Figure 6). The recombination bins were judged as described in Huang et al.[67], using the genome-resequencing data of HHZ, P28, and H471 (unpublished data).

To evaluate the DT performance of the three genotypes, a pot experiment was arranged in a randomized complete block design with three treatments (well-watered, 1-day drought stressed, and 3-day drought stressed) and six replications or pots in a greenhouse at the Institute of Crop Sciences of Chinese Academy of Agricultural Sciences (Beijing, China). The days were counted after the available water content (AWC) in the soil reached 20% to allow drought measurements at precisely determined intervals. Three healthy seedlings of the three genotypes were transplanted equidistantly into a strip pot (15 cm in height and 50 cm in diameter) filled with 2 kg of sterilized field soil, which contained about 50% AWC of the soil, as measured by soil moisture meters (TZS-W, Zhejiang Top Instrument Co. Ltd). Seedlings of each genotype were planted in six pots giving a total of six plants; all plants were grown with 14 h daylight at 28°C and a 10-h dark period at 25°C under controlled conditions. Withholding water at the tillering stage started the drought stress treatment. The soil water content reached 15%, 10%, and 7.5% after 1 day, 3 days, and 4 days of drought treatment, respectively.

Yield performance was evaluated in experiments under drought stress and well-watered conditions at the experimental farm of International Rice Research Institute (Philippines). Seedlings of three genotypes were transplanted into a three-row plot with 45 plants per plot at a spacing of 15 × 25 cm between rows and plants within each plot; three replications for each genotype. For drought stress treatment, water was drained and irrigation was held at the peak tillering stage until maturity. In the well-watered control, the field was irrigated at weekly intervals until 2 weeks before harvesting. Grain yield was measured on the 10 plants sampled at maturity from the middle row of each plot.

### SEM analysis

The second leaves from 1-day drought-stressed plants grown in the pots were used for SEM analyses, as described in You et al.[68] with minor modifications. Fresh leaf samples were pre-fixed for 3 h in 3% glutaraldehyde-sodium phosphate buffer (0.1 M) at room temperature and rinsed three times with 0.1 M sodium phosphate buffer. Post-fixation was performed with 2% OsO4 at 4°C. The samples were dehydrated through an ethanol series and infiltrated with an isoamyl acetate series. The samples were then coated with metal particles for analysis by SEM to observe the guard cells. A Hitachi S750 scanning electron microscope (http://www.hitachi-hitec.com/global/em/) was used to take photographs, and the numbers of guard cells in randomly chosen fields were counted and analyzed statistically.

### Physiological traits of the three genotypes under drought stress

Detached leaves are weighed and saturated with water for 24 h, then weighed again and dried for 48 h, and weighed again. RWC was calculated using the following formula: RWC (%) = [(FM - DM)/(TM - DM)] × 100, where FM, DM, and TM are the fresh, dry, and turgid masses of the tissue weighed, respectively. Monitoring the fresh weight loss at the indicated time points (per hour) measured the WLRs of detached leaves[68]. Measuring solute leakage from rice leaf tissue evaluated the REL, according to the method of Arora et al.[69], with minor modifications. Three replicates of 0.5 g fresh leaves were sampled from control and drought-treated plants. After being cut into 1-cm pieces, the 0.5 g leaf samples were immersed in 20 mL distilled water in a test tube for 1 h with the help of a vacuum pump. After standing for 2 h at 25°C, water conductivity was measured. Leaf discs were then killed in the same solution by autoclaving, and total conductivity was measured at room temperature. Percent injury arising from each treatment was calculated from the conductivity data using the equation: % injury = [(% L(t) - % L(c))/(100 - % L(c))] × 100), where % L (t) and % L(c) are percent conductivity for treated and control samples, respectively. Antioxidant enzyme activity, including catalase (CAT) and ascorbate peroxidase (APX), were determined following previously reported methods[70].

Enzyme-linked immuno-sorbent assay (ELISA) was performed to measure JA content. Three replicates of 200 ~ 500 mg fresh leaves were sampled from control and drought-stressed plants and immediately ground with 80% methanol under ice-bath conditions, endogenous JA content was measured according to the manufacture’s protocol (RB Plant-JA, Cat No.DRE-P10695, USA).

### RNA extraction, RNA-seq library construction, and sequencing

Three top leaves for each sample (two replicates for each sample) were harvested for each genotype under 1 day and 3 days of drought stress and under well-watered control conditions. All samples were immediately frozen in liquid nitrogen and stored at -80°C. The TRIzol Reagent (Invitrogen, USA) was used to extract total RNA, which was quantified by a Qubit RNA assay kit (Applied Biosystems, CA, USA). An Agilent 2100 Bioanalyzer (Agilent Technologies) was used to check the RNA integrity. The ribosomal RNA (rRNA) was removed from 8 μg of total RNA using a RiboMinus™ Plant Kit (Invitrogen), followed by a Ribo-Zero Gram-Negative Bacteria kit (Epicentre), according to the manufacturer’s instructions. The TruSeq RNA Sample Preparation kit (Illumina) was used to construct the paired-end fragment library, with minor modifications. Briefly, the rRNA removed RNA was fragmented and the first-strand cDNA was synthesized using random hexamers and SuperScript II Reverse Transcriptase. The RNA template was then removed and a replacement strand was synthesized to generate double-stranded (ds) cDNA. After end repair and 3′ end adenylation, an indexed adapter was ligated to the dsDNA. Fragments of 300–350 bp were excised and enriched by 12 cycles of PCR. The QUBIT and Agilent 2100 Bioanalyzer, assessed the yield and size distribution of the PCR products, respectively. CapitalBio Corporation, Beijing, China subjected the produced libraries to cluster generation on cBot and sequencing on a HiSeq 2000 platform (Illumina) with paired-end 100 base pair reads. The Illumina instrument software performed primary data analysis and base calling. Raw sequence data are available in the NCBI’s Gene Expression Omnibus (GEO) database under the accession number GSE57950.

### Transcriptome data analysis

An in-house perl script was used to remove adaptor sequences and low-quality sequences from the raw reads. The retained high-quality pair-end reads of rice for each sample were mapped to the rice genome of RGAP at MSU[71] by TopHat[72] and then assembled using Cufflinks[73] to construct unique transcripts sequences, using the parameter: -g -b -u -o. Cuffcompare[73] was used to compare the assembled transcript fragments of each sample to the reference annotation, constructing a non-redundant transcripts data set among the samples. The number of mapped clean reads for each gene was counted and normalized into the reads per kilo base per million value[74]; Cuffdiff[73] was then used to identify DEGs. Finally, genes with a *p-*value ≤0.001 were designated as significantly differential expressed between each pair of samples.

Gene function annotations were performed based on the Rice Genome Annotation Project version 7[71]. AgriGO was used to perform GO enrichment analysis[75]. The Kyoto encyclopedia of genes and genomes (KEGG) pathway enrichment was performed using a hypergeometric test. The analysis incorporated false discovery rate correction using the Benjamini and Hochberg method of multiple hypotheses testing to reduce false negatives[76]. A weighted gene co-expression network analysis[77] was used to construct gene coexpression networks based on a Pearson correlation coefficient >0.75.

### Quantitative real-time reverse transcription-PCR (qRT-PCR) analysis

To validate the results of the Illumina sequencing experiment, an independent set of samples (three biological replicates per sample) were collected as described for the RNA sequencing. Total RNA was treated with DNase I (TransGene, Beijing, China) to remove residual genome DNA and cDNA synthesis was performed using EasyScript First-Strand cDNA Synthesis SuperMix (TransGene, Beijing, China) according to the manufacturer’s protocol. qRT-PCR was used to verify a subset of DEGs, using the methods described by Swarbrick et al.[78]. The gene sequences were downloaded from the rice genome of RGAP at MSU[71]; Primer 3 software (http://frodo.wi.mit.edu/) was used to design the primers (Additional file3). Twenty-seven rice genes with various function categories were selected and tested in 20-μL reactions using the SYBR® Green PCR Master Mix kit (Applied Biosystems, CA, USA), following the manufacturer’s protocol, via an ABI Prism 7900 Sequence Detection System (Applied Biosystems). The relative expression of each gene was calculated according to the method of 2^-△△Ct^[79]. The *Actin 1* gene (LOC_Os03g50890) was used as endogenous references for qRT-PCR, and all analyses were performed with three technical and three biological replicates.

## Electronic supplementary material

Additional file 1:**Hierarchical cluster analysis of nine sample pools (columns) and all expressed genes, under control and drought stress conditions (rows).** A PowerPoint file containing hierarchical cluster analysis of nine sample pools (columns) and all expressed genes, under control and drought stress conditions (rows). The raw data represented here can be obtained from GEO: GSE57950. In the colored panels, each horizontal line represents a single gene and the colored line indicates the expression level (in a log scale) of the gene relative to the median in a specific sample: high expression in red, low expression in green. (PPT 182 KB)

Additional file 2:**Correlation analysis of Illumina sequencing results between two replicates of each sample for H471, P28, and HHZ, under control (ck), 1 day and 3 days of drought stress, respectively.** A PowerPoint file containing correlation analysis of Illumina sequencing results between two replicates of each sample for H471, P28, and HHZ, under control (ck), 1 day and 3 days of drought stress, respectively. (PPT 295 KB)

Additional file 3:**Information of primers used in qRT-PCR analysis.** Excel file containing information of primers used in qRT-PCR analysis. (XLS 22 KB)

Additional file 4:**Comparison of transcription measurements by Illumina sequencing and quantitative real-time reverse transcription-PCR (qRT-PCR) assays.** A PowerPoint file containing comparison of transcription measurements by Illumina sequencing and quantitative real-time reverse transcription-PCR (qRT-PCR) assays. (A) The correlation coefficient (*R2*) between the two datasets is 0.93. (B) Comparative analysis of six candidate genes expression level by qRT-PCR and RNA-seq. qRT-PCR quantification values were compared with HHZ_ck. Error bars indicate the standard deviation. *Actin 1* was used as an endogenous control. (PPT 800 KB)

Additional file 5:**DEGs in P28 and H471 compared with HHZ under a well-watered condition.** Excel file containing a list of DEGs in P28 and H471 compared with HHZ under a well-watered condition. (XLS 338 KB)

Additional file 6:**GO enrichment analysis of DEGs in various groups.** Excel file containing five lists of GO enrichment of DEGs in various groups: 6–1, GO enrichment analysis of genes with the higher basal expression in H471 compared with HHZ; 6–2, GO enrichment analysis of the commonly up-regulated genes in three genotypes under 1 day of drought stress; 6–3, GO enrichment analysis of the commonly down-regulated genes under 1 day of drought stress; 6–4, GO enrichment analysis of the commonly up-regulated genes in three genotypes under 3 days of drought stress; 6–5, GO enrichment of the commonly down-regulated genes in three genotypes under 3 days of drought stress (XLS 47 kb). (XLS 47 KB)

Additional file 7:**Genes list of higher basal expression in P28 and H471 compared with HHZ.** Description: Excel file containing five list of genes with higher basal expression in P28 and H471 compared with HHZ. (XLS 337 KB)

Additional file 8:**DEGs list in H471 compared with HHZ under drought stress.** Excel file containing a list of DEGs in H471 compared with HHZ under drought stress. (XLS 158 KB)

Additional file 9:**List of DEGs with various function categories in H471 compared with HHZ under drought conditions.** Excel file containing a list of DEGs with various function categories in H471 compared with HHZ under drought conditions. (XLS 57 KB)

Additional file 10:**List of alpha-linolenic acid metabolism-related genes in H471 vs. HHZ DEGs.** Excel file containing a list of alpha-linolenic acid metabolism-related genes in H471 vs. HHZ DEGs. (XLS 51 KB)

Additional file 11:**Validation of JA related genes by qRT-PCR.** A PowerPoint file containing comparison validation of eight JA related genes by qRT-PCR assay. qRT-PCR Quantification values were compared with HHZ_ck. Error bars indicate the standard deviation. *Actin 1* was used as an endogenous control. (PPT 924 KB)

Additional file 12:**Information on introgressed chromosome segments and the differentially expressed genes in H471 compared with HHZ under control and drought stress conditions and their corresponding DT-related QTLs.** Excel file containing information on introgressed chromosome segments and the differentially expressed genes in H471 compared with HHZ under control and drought stress conditions and their corresponding DT-related QTLs. (XLS 61 KB)
