# Supplementary material for: Comparative transcriptome sequencing of tolerant rice introgression line and its parents in response to drought stress
Source: BMC Genomics. 2014 Nov 26;15(1):1026. doi: 10.1186/1471-2164-15-1026 (PMC4258296; doi:10.1186/1471-2164-15-1026)
Supplement: Supplementary file 1 — Additional file 1:Hierarchical cluster analysis of nine sample pools (columns) and all expressed genes, under control and drought stress conditions (rows). A PowerPoint file containing hierarchical cluster analysis of nine sample pools (columns) and all expressed genes, under control and drought stress conditions (rows). The raw data represented here can be obtained from GEO: GSE57950. In the colored panels, each horizontal line represents a single gene and the colored line indicates the expression level (in a log scale) of the gene relative to the median in a specific sample: high expression in red, low expression in green. (PPT 182 KB) [file 12864_2014_6721_MOESM1_ESM.ppt]

## Slide 1
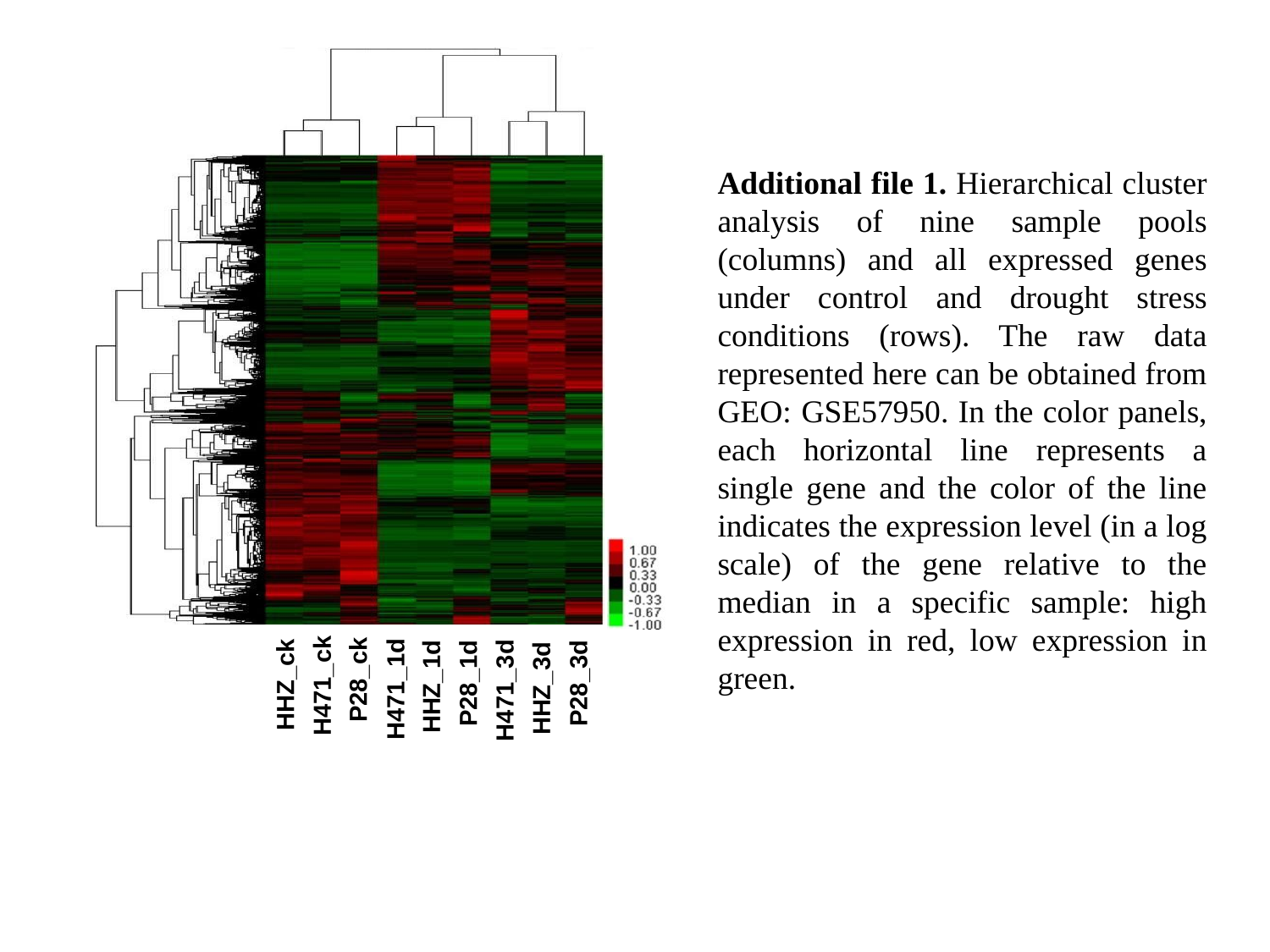

P28_ck
P28_1d
P28_3d
HHZ_ck
H471_ck
HHZ_1d
HHZ_3d
H471_1d
H471_3d
Additional file 1. Hierarchical cluster analysis of nine sample pools (columns) and all expressed genes under control and drought stress conditions (rows). The raw data represented here can be obtained from GEO: GSE57950. In the color panels, each horizontal line represents a single gene and the color of the line indicates the expression level (in a log scale) of the gene relative to the median in a specific sample: high expression in red, low expression in green.
