# Supplementary material for: Comparative transcriptome sequencing of tolerant rice introgression line and its parents in response to drought stress
Source: BMC Genomics. 2014 Nov 26;15(1):1026. doi: 10.1186/1471-2164-15-1026 (PMC4258296; doi:10.1186/1471-2164-15-1026)
Supplement: Supplementary file 2 — Additional file 2:Correlation analysis of Illumina sequencing results between two replicates of each sample for H471, P28, and HHZ, under control (ck), 1 day and 3 days of drought stress, respectively. A PowerPoint file containing correlation analysis of Illumina sequencing results between two replicates of each sample for H471, P28, and HHZ, under control (ck), 1 day and 3 days of drought stress, respectively. (PPT 295 KB) [file 12864_2014_6721_MOESM2_ESM.ppt]

## Slide 1
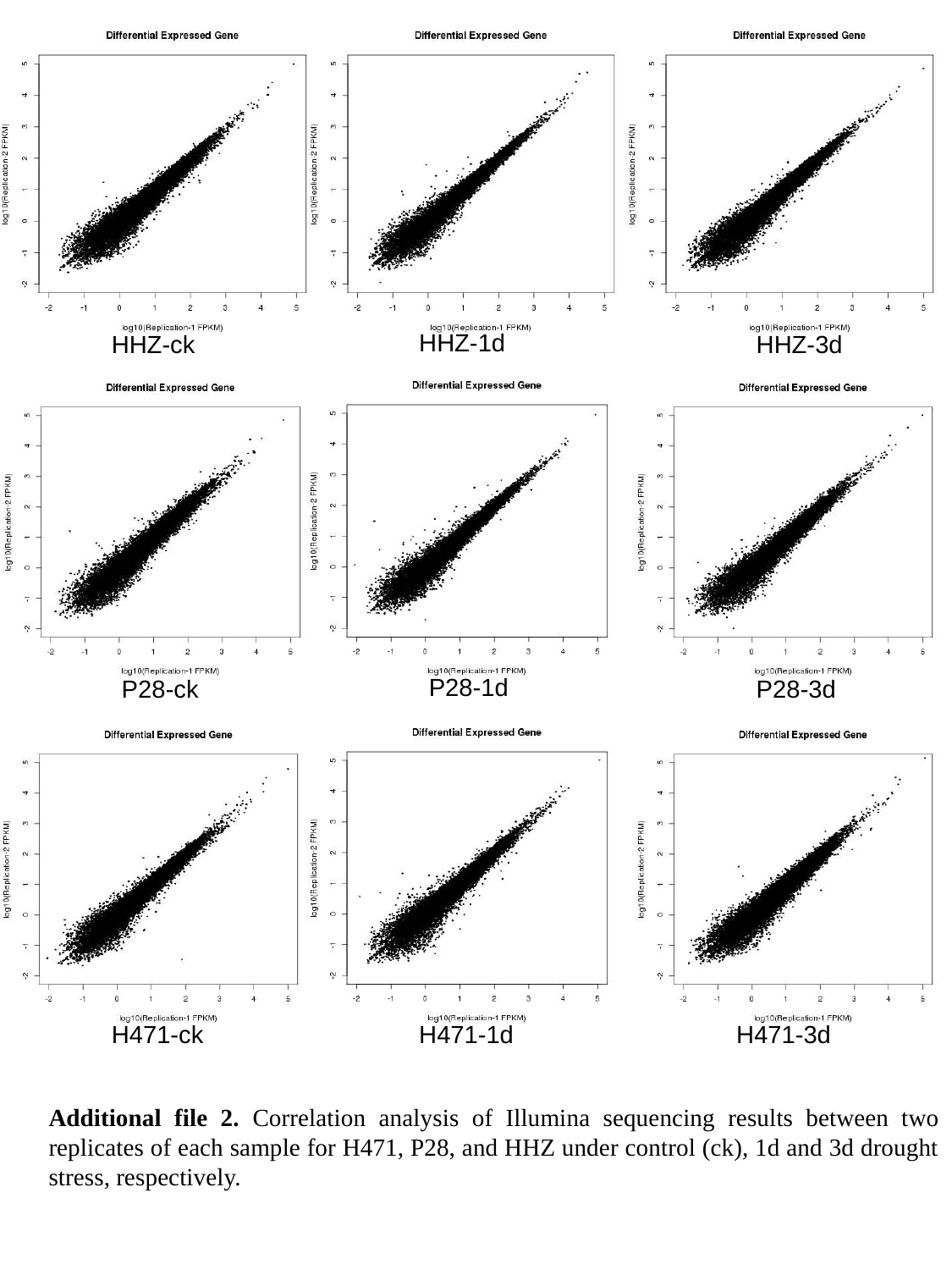

HHZ-1d
HHZ-ck
HHZ-3d
P28-1d
P28-ck
P28-3d
H471-ck
H471-1d
H471-3d
Additional file 2. Correlation analysis of Illumina sequencing results between two replicates of each sample for H471, P28, and HHZ under control (ck), 1d and 3d drought stress, respectively.
