# Supplementary material for: Comparative transcriptome sequencing of tolerant rice introgression line and its parents in response to drought stress
Source: BMC Genomics. 2014 Nov 26;15(1):1026. doi: 10.1186/1471-2164-15-1026 (PMC4258296; doi:10.1186/1471-2164-15-1026)
Supplement: Supplementary file 4 — Additional file 4:Comparison of transcription measurements by Illumina sequencing and quantitative real-time reverse transcription-PCR (qRT-PCR) assays. A PowerPoint file containing comparison of transcription measurements by Illumina sequencing and quantitative real-time reverse transcription-PCR (qRT-PCR) assays. (A) The correlation coefficient (R2) between the two datasets is 0.93. (B) Comparative analysis of six candidate genes expression level by qRT-PCR and RNA-seq. qRT-PCR quantification values were compared with HHZ_ck. Error bars indicate the standard deviation. Actin 1 was used as an endogenous control. (PPT 800 KB) [file 12864_2014_6721_MOESM4_ESM.ppt]

## Slide 1
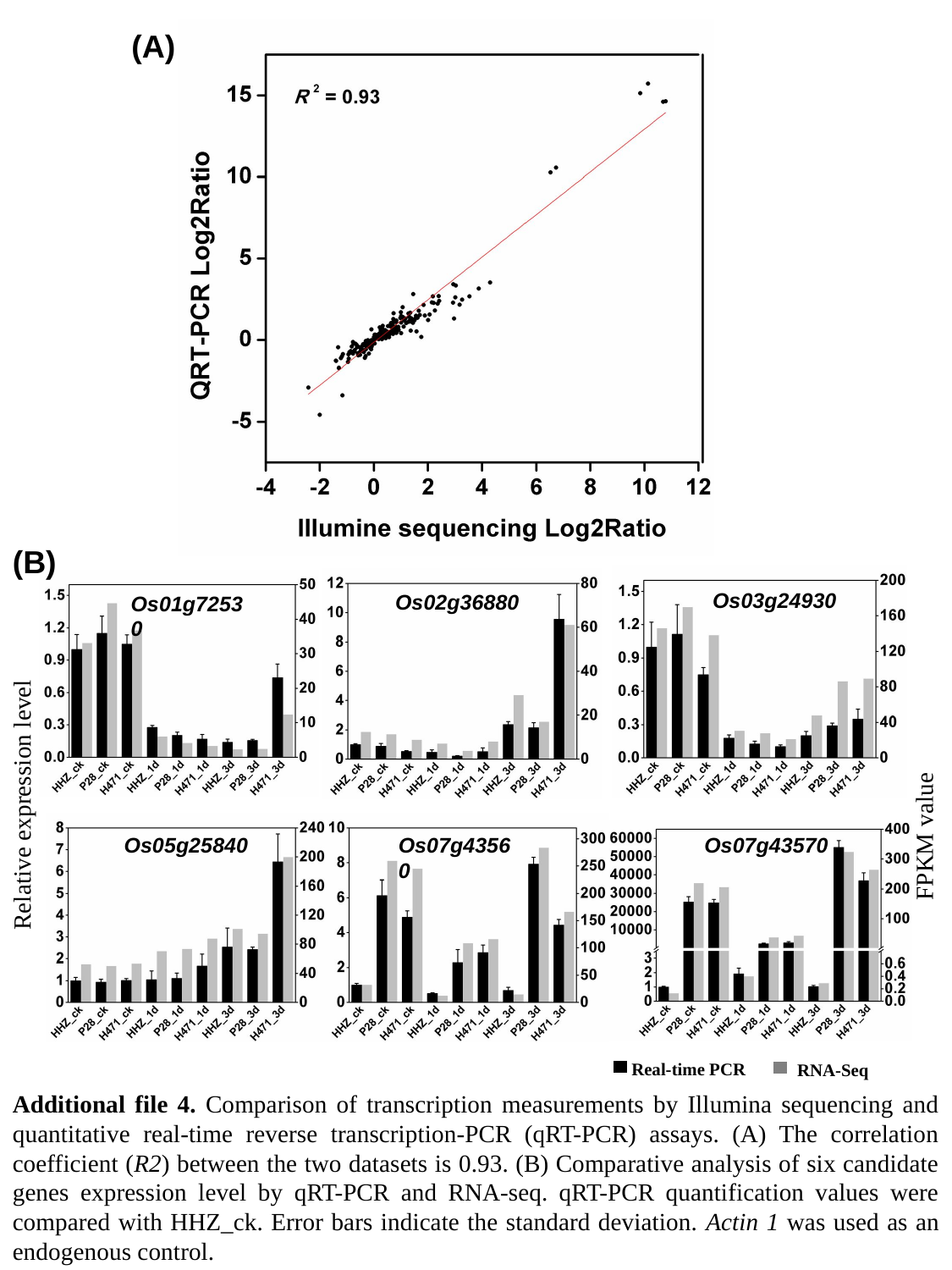

(A)
Os03g24930
Os02g36880
Os01g72530
Os05g25840
Os07g43560
Os07g43570
Relative expression level
FPKM value
Real-time PCR
RNA-Seq
(B)
Additional file 4. Comparison of transcription measurements by Illumina sequencing and quantitative real-time reverse transcription-PCR (qRT-PCR) assays. (A) The correlation coefficient (R2) between the two datasets is 0.93. (B) Comparative analysis of six candidate genes expression level by qRT-PCR and RNA-seq. qRT-PCR quantification values were compared with HHZ_ck. Error bars indicate the standard deviation. Actin 1 was used as an endogenous control.
