# Supplementary material for: Comparative transcriptome sequencing of tolerant rice introgression line and its parents in response to drought stress
Source: BMC Genomics. 2014 Nov 26;15(1):1026. doi: 10.1186/1471-2164-15-1026 (PMC4258296; doi:10.1186/1471-2164-15-1026)
Supplement: Supplementary file 11 — Additional file 11:Validation of JA related genes by qRT-PCR. A PowerPoint file containing comparison validation of eight JA related genes by qRT-PCR assay. qRT-PCR Quantification values were compared with HHZ_ck. Error bars indicate the standard deviation. Actin 1 was used as an endogenous control. (PPT 924 KB) [file 12864_2014_6721_MOESM11_ESM.ppt]

## Slide 1
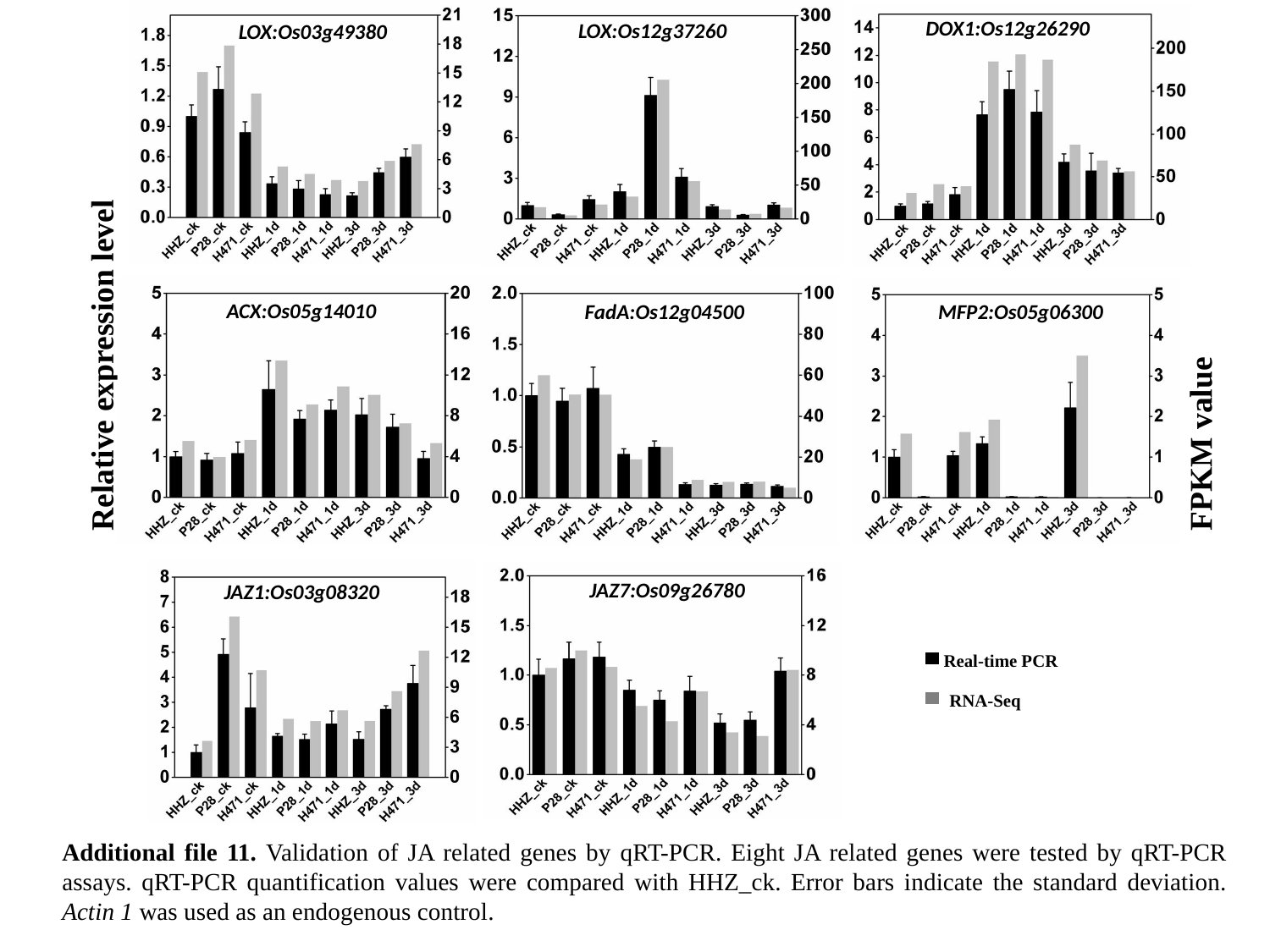

LOX:Os03g49380
LOX:Os12g37260
DOX1:Os12g26290
ACX:Os05g14010
FadA:Os12g04500
MFP2:Os05g06300
Relative expression level
FPKM value
JAZ1:Os03g08320
JAZ7:Os09g26780
Real-time PCR
RNA-Seq
Additional file 11. Validation of JA related genes by qRT-PCR. Eight JA related genes were tested by qRT-PCR assays. qRT-PCR quantification values were compared with HHZ_ck. Error bars indicate the standard deviation. Actin 1 was used as an endogenous control.
